# Aphasien bei lakunären Hirninfarkten

**DOI:** 10.1007/s00115-021-01072-6

**Published:** 2021-02-16

**Authors:** Konstantin Kohlhase, Jan Hendrik Schaefer, Sriramya Lapa, Alina Jurcoane, Marlies Wagner, Pavel Hok, Christian A. Kell

**Affiliations:** 1 Klinik für Neurologie, Goethe-Universität Frankfurt, Schleusenweg 2–16, 60528 Frankfurt, Deutschland; 2 Institut für Neuroradiologie, Goethe-Universität Frankfurt, Frankfurt, Deutschland; 3 Klinik für Neurologie, Universitätskrankenhaus Olomouc und Fakultät der Medizin und Zahnmedizin, Palacký Universität, Olomouc, Tschechien

**Keywords:** Hirninfarkt, Lakunär, Subkortikal, Aphasie, Fasciculus arcuatus, Ischemic stroke, Lacunar, Subcortical, Aphasia, Arcuate fascicle

## Abstract

**Hintergrund:**

Aphasien gehören nicht zu den typischen klinischen Manifestationen lakunärer Hirninfarkte, sind jedoch im Rahmen seltener atypischer lakunärer Syndrome beschrieben.

**Ziel der Arbeit:**

Beschreibung von Aphasiemustern und betroffener Fasertrakte bei lakunären Infarkten.

**Material und Methoden:**

Fallserie von drei Patienten mit in der Magnetresonanztomographie nachgewiesenen lakunären Hirninfarkten und Aphasie. Identifikation betroffener Faserbahnen mittels Fasertraktographie der koregistrierten Schädigungsorte in Gehirnen zweier gesunder Probanden.

**Ergebnisse:**

Radiologisch waren die Lakunen, die Aphasien hervorriefen, weit lateral im Marklager der linken Hemisphäre gelegen und befanden sich im Vergleich zu der Lakune eines nichtaphasischen Kontrollpatienten weiter rostrodorsal. Klinisch fand sich trotz Aussparung des Kortex, Thalamus und weiter Teile der Basalganglien eine leichte bis moderate nichtflüssige Aphasie mit syntaktischen Defiziten. In der Fasertraktographie zeigten die aphasischen im Vergleich zum nichtaphasischen Patienten eine stärkere Affektion der Fasern des linken Fasciculus arcuatus sowie eine Beteiligung des frontostriatalen und frontalen Aslant-Trakts.

**Diskussion:**

Links lateral gelegene lakunäre Infarkte können durch Beteiligung sprachrelevanter Fasertrakte eine klinisch relevante Aphasie hervorrufen.

## Abstract (en)

**Background:**

Typical lacunar syndromes do not include aphasia but aphasia has been reported in rare atypical lacunar syndromes.

**Objective:**

Description of the phenomenology and of affected fiber tracts.

**Material and methods:**

Case series of three patients with lacunar stroke as evidenced by magnetic resonance imaging. Identification of affected fiber tracts via fiber tracking from coregistered lesion sites in brains of two healthy participants.

**Results:**

The lacunar strokes that produced aphasia were located in the very lateral territory of perforating branches of the middle cerebral artery and extended along the external capsule into its most rostrodorsal aspect. Even though the cortex, thalamus and most parts of the basal ganglia were unaffected, patients exhibited a mild to moderate nonfluent aphasia with syntactic deficits. Fiber tracking revealed that in contrast to the nonaphasic control patient with a neighboring lacunar stroke, the aphasic patient strokes involved particularly fibers of the left arcuate fascicle as well as fibers of the frontostriatal and frontal aslant tracts.

**Conclusion:**

Left lateral lacunar stroke can cause clinically relevant aphasia through disruption of speech-relevant fiber tracts.

Eine Aphasie gilt generell als kortikale Funktionsstörung, die in der Regel nicht mit lakunären Infarzierungen außerhalb der Basalganglien und des Thalamus vereinbar ist. Anhand von drei hiervon abweichenden Fallbeispielen aus unserer klinischen Routine auf der Schlaganfallstation und mithilfe der Magnetresonanztomographie zur Fasertraktanalyse untersuchten wir, welche mit der Sprachgenerierung assoziierten Bahnsysteme einer Aphasie bei lakunären Schlaganfällen zugrunde liegen können. Zudem wurden die klinischen Schädigungsmuster analysiert.

## Hintergrund

In der akutmedizinischen Schlaganfallversorgung, in der Grundsätze wie „time is brain“ den zeitlichen Aspekt der Versorgung in den Vordergrund stellen, ist die schnelle Kategorisierung des klinischen Syndroms von entscheidender Bedeutung. Um einen Verschluss eines proximalen hirnversorgenden Gefäßes, der einer mechanischen Rekanalisation zugänglich wäre, von einem mikroangiopathischen Infarkt zu unterscheiden, hilft in der klinisch-neurologischen Untersuchung die Suche nach kortikalen Zeichen wie Apraxie, Neglekt oder Aphasie. Lakunäre Infarkte auf dem Boden von Perforatorenverschlüssen führen laut geltender Lehrbuchmeinung daher in der Regel zu typischen lakunären Syndromen, welche keine Aphasie oder andere kortikale Zeichen beinhalten [9]. Dabei ist das Phänomen einer subkortikalen Aphasie kein unbekanntes und wurde zuerst bei größeren Läsionen im Bereich der striatokapsulären Strukturen mit Beteiligung des periventrikulären Marklagers [1, 8] oder des Thalamus [11, 13] beschrieben. Den publizierten Fällen lagen zum Teil große Läsionen zugrunde, sodass mehrere subkortikale Strukturen wie die Basalganglien, Assoziationsbahnen oder der Thalamus betroffen waren und somit der Beitrag einzelner subkortikaler Areale zur Aphasie nicht zu klären war. Auch konnte insbesondere in den älteren Fallbeschreibungen in Ermangelung einer zerebralen Magnetresonanztomographie (cMRT) eine zusätzliche Beteiligung kortikaler Areale nicht ausgeschlossen werden. Vereinzelt sind Aphasien als Manifestation seltener atypischer lakunärer Syndrome beschrieben und Autoren schlugen unterschiedliche Erklärungsansätze vor, ohne dass bislang eine konsistente Läsions-Ausfalls-Beziehung identifiziert werden konnte [2, 12, 26].

Anhand von drei Kasuistiken möchten wir Ausnahmen demonstrieren, welche der klassischen klinischen Syndromeinteilung zu widersprechen scheinen und anhand derer wir einen Erklärungsansatz für eine Aphasie auf dem Boden eines lakunären Infarkts liefern möchten. Im Gegensatz zu vorangegangenen Studien, die sich mit subkortikalen Aphasien beschäftigten, war nachgewiesenermaßen in der cMRT nur ein kleiner Teil striatokapsulärer Strukturen betroffen, wohingegen der Thalamus und weite Teile der Basalganglien ausgespart waren.

## Methoden

### Logopädische Testung

In der Akutphase des Hirninfarkts erfolgte eine klinisch-logopädische Untersuchung, die die Spontansprache sowie das Vorliegen einer Dysarthrie oder Sprechapraxie überprüfte. Erstere wurde bei Auffälligkeiten der Phonation oder Artikulation diagnostiziert, letztere war definiert durch Suchbewegungen der Artikulationsorgane, Sprechanstrengung und Selbstkorrektur [23]. Bei Vorliegen einer Aphasie folgte die Durchführung eines zusätzlichen Aachener Aphasietests (AAT) außerhalb der Aufnahmesituation [14]. Die Sprachflüssigkeit wurde insbesondere anhand der Spontansprache (Antwort auf offene Fragen) bewertet, wobei eine Störung des Redeflusses mit einer Geschwindigkeit von weniger als 50 Wörtern pro Minute mit Sprechpausen als nichtflüssige Aphasie gewertet wurde [15]. Zusätzlich wurde auf Satzebene die Komplexität der Syntax z. B. anhand des Gebrauchs von Attributen und Nebensätzen beurteilt.

### cMRT

**Figure 1:**
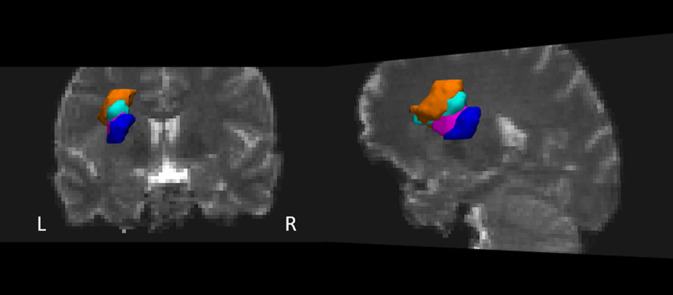

Im Rahmen der Routineversorgung erhielten alle Patienten eine standardisierte klinisch-neurologische Untersuchung, gefolgt von einer zerebralen Bildgebung mittels zerebraler 3T-cMRT (Skyra, Siemens, München, Deutschland) inklusive 3‑D-Sequenzen und Time-of-flight-Angiographie. Eine diffusionsgewichtete Standardsequenz diente zur Ischämiedetektion (Ischämienachweis im Stromgebiet einer einzelnen Perforatorarterie mit einer Größe < 15–20 mm [25]). Die ischämischen Areale wurden auf dem b0-Bild einer 3‑D-Diffusionstensorsequenz (40 nichtkollineare Gradientenrichtungen; b‑Wert 700 s/mm^2^, TR 9200 ms, TE 72 ms, Voxeldimensionen 1,9 × 1,9 × 1,9 mm, 50 transversale Schichten) manuell markiert (itk-SNAP; www.itksnap.org; University of Pennsylvania, Philadelphia, PA, USA) und mithilfe einer 3‑D-T1-gewichteten Sequenz koregistriert („axial magnetization prepared rapid gradient echo“; TR 2300 ms, TE 2,32 ms, TI 900 ms, Flip-Winkel 8°, 192 sagittale Schichten, Voxeldimensionen 0,9 mm^3^). Die Berechnung einer affinen Transformation mit 12 Freiheitsgraden zwischen der Diffusions- und T1-gewichteten Sequenz erfolgte mithilfe des FMRIB Linear Image Registration Tool (University of Oxford, Vereinigtes Königreich) [17, 18]. Die T1-gewichteten Bilder wurden mittels FMRIB Nonlinear Image Registration Tool in den MNI-152-Standard-Raum übertragen und die Transformationsparameter auf die Diffusionsbilder mitsamt Masken übertragen. Die koregistrierten Lakunen sind in Abb. 1 auf dem b0-Bild eines gesunden Probanden dargestellt.

**Figure 2:**
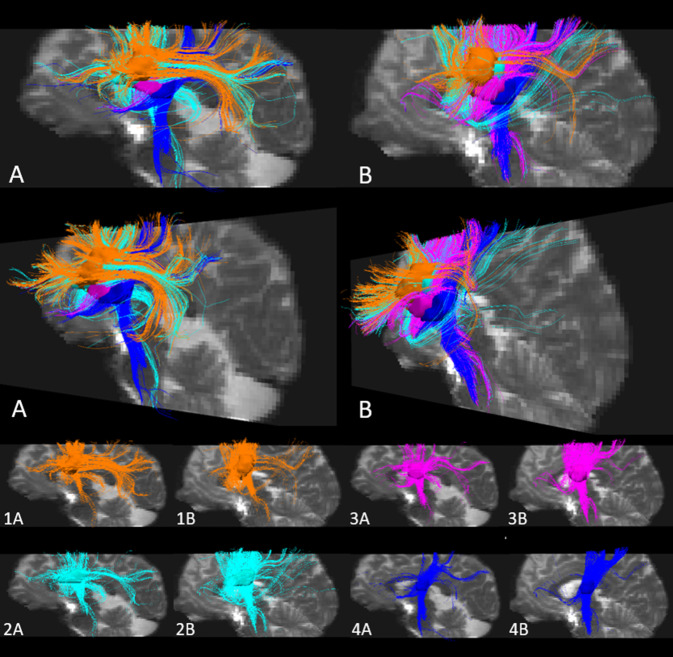

**Figure 3:**
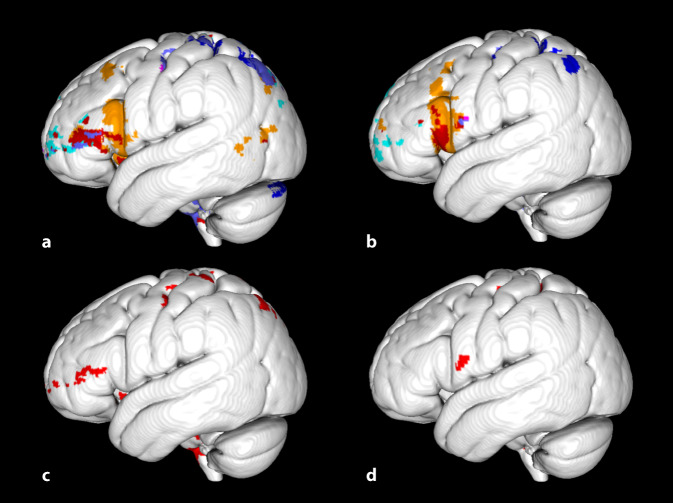

Zur Untersuchung, welche Faserbahnen durch die Lakunen betroffen waren, führten wir Fasertraktographien durch. Da die Fasertraktographie bei Patienten durch die strukturelle Läsion verfälscht werden kann, identifizierten wir die Faserbahnen bei zwei gesunden Probanden nach Koregistrierung der Gehirne mit den Gehirnen der Patienten, ausgehend von dem Ort der Lakunen der Patienten. Der Traktographiealgorithmus in DSI-Studio (http://dsi-studio.labsolver.org; University of Pittsburgh, PA, USA) verfolgt deterministisch Fasern mit quantitativer Anisotropie als Abbruchkriterium [27]. Darstellungen der Fasertrakte in Abb. 2 wurden mittels TrackVis (http://trackvis.org/; University of Pittsburgh, PA, USA) erstellt. Zur Identifizierung der kortikalen Ursprungs- und Zielregionen der Faserbahnen erfolgte eine probabilistische Fasertraktographie mithilfe der FMRIB Diffusion Toolbox Version 5.0.10 (FSL, www.fmrib.ox.ac.uk/fsl; University of Oxford, Vereinigtes Königreich [19, 24]). Die Standardverarbeitung der Daten umfasste eine Korrektur für „eddy currents“ und Kopfbewegung sowie eine Hirnextraktion. Die Wahrscheinlichkeitsverteilung der Diffusionsparameter und der Faserverläufe für jedes einzelne Voxel wurde mittels des Markov-Chain-Monte-Carlo-Verfahrens in BEDPOSTX geschätzt [4–6]. Die probabilistische Traktographie wurde für jede Maske getrennt in PROBTRACKX [6] durchgeführt. Pro Voxel einer Maske im Standardraum wurden 5000 Verläufe (Wahrscheinlichkeitsgradienten in jeweils entgegengesetzten Richtungen) erzeugt. Ein Abbruch des Trackings erfolgte nach entweder 2000 0,5-mm-Schritten, einem Winkel von > 78,5° zwischen zwei nacheinander folgenden Schritten oder bei Verlassen der Gehirnmaske. Auf der Basis dieser Datenpunkte wurde eine Karte der Konnektivitätsverteilung (oder Faserdichte) erzeugt. Jede einzelne Karte wurde durch die Anzahl aller verbliebenen Faserverläufe geteilt, sodass der Wert eines Voxels die relative Wahrscheinlichkeit angibt, dass ein Fasertrakt den Voxel mit der Maske verbindet, den sog. Konnektivitätsindex [16]. Die hieraus resultierende Karte wurde mit einem Schwellenwert an der 90. Perzentile binarisiert. Diese Konnektivitätskarten wurden zur Illustration der kortikalen Projektionen in Abb. 3 mittels Mango 4.0.1 (http://rii.uthscsa.edu/mango; University of Texas, San Antonio, TX, USA) auf einem MNI-152-Standard-Gehirn dargestellt (5 mm Suchtiefe).

Um die Lakunen der aphasischen Patienten und deren Faserverbindungen mit einem nichtaphasischen Kontrollpatienten zu vergleichen, schlossen wir einen Patienten in unsere Studie ein, der im Studienverlauf die nächstgelegene Lakune zu den Lakunen der aphasischen Patienten aufwies. Da die Lakune des nichtaphasischen Kontrollpatienten teilweise mit der Lakune der aphasischen Patientin 3 überlappte, erstellten wir eine zusätzliche Maske zur Traktographie, die der Differenz zwischen der Lakune der aphasischen Patientin 3 und dem nichtaphasischen Kontrollpatienten entsprach. Die strukturelle Konnektivität dieser Maske sollte kortikale Projektionsfelder aphasiekritischer Faserbahnen aufdecken.

## Ergebnisse

### Fall 1

**Figure 4:**
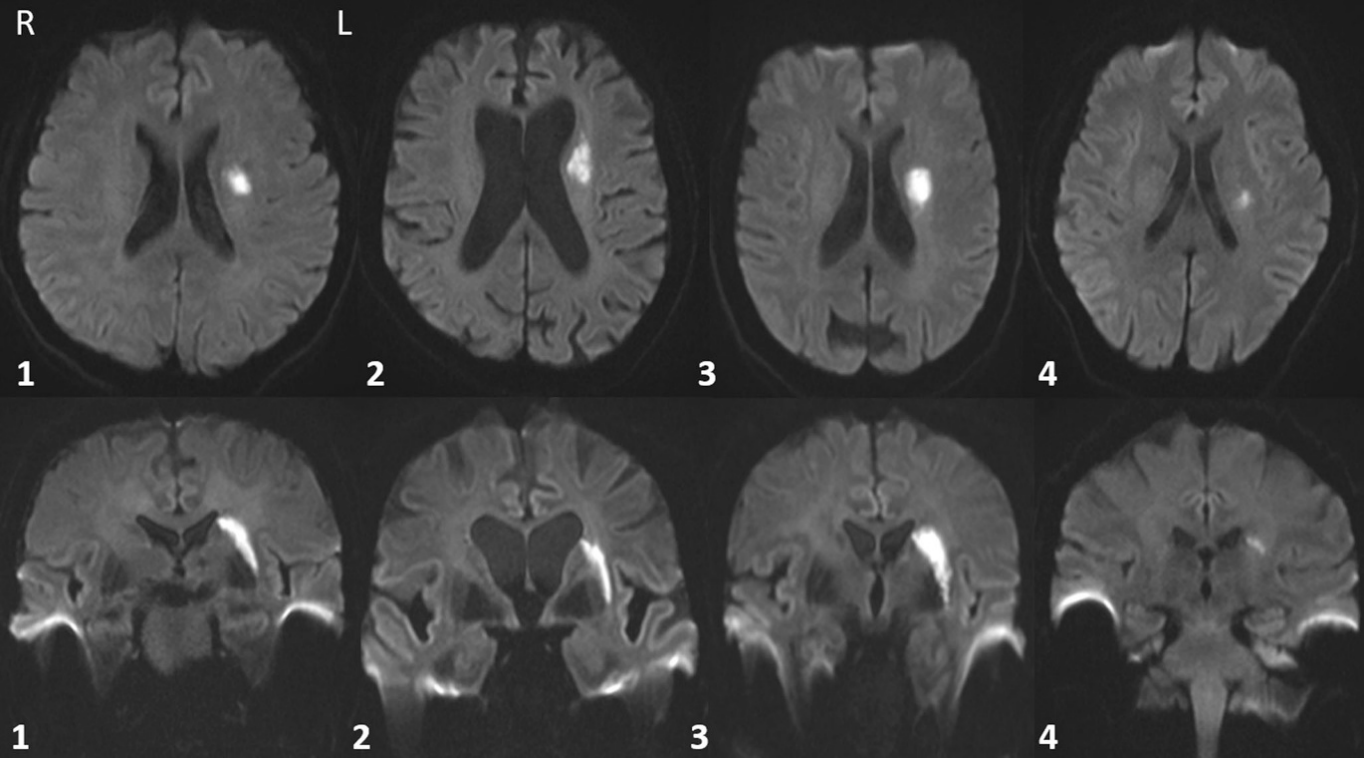

Ein 62-jähriger Deutschlehrer stellte sich mit einer seit drei Tagen bestehenden Gangstörung vor, aufgrund derer er nicht mehr habe Treppen steigen können, da er mit seinen Füßen an den Stufen hängengeblieben sei. Zusätzlich sei ihm eine Wortfindungsstörung sowohl für seine Muttersprache als auch für die deutsche Sprache aufgefallen, die er in der Jugend erlernt hatte und für die er eigenanamnestisch keine prämorbiden Auffälligkeiten zeigte. Die Schwierigkeiten habe er v. a. beim Verfassen von E‑Mails bemerkt. In der durchgeführten cMRT zeigte sich ein lakunärer Infarkt in der linken Capsula externa, der das externe Pallidumsegment tangierte und sich bis in das frontale Marklager erstreckte (Abb. 4, Patient 1).

In der logopädischen Untersuchung zeigten sich Defizite des Sprachverständnisses bei der Verarbeitung komplexer Syntax. Die spontane Sprachflüssigkeit war vermindert mit einer vereinfachten Satzstruktur, ohne dass grammatikalische Fehler auftraten. Dies zeigte sich auch beim Benennen, wohingegen das Benennen auf Wortebene regelrecht war. Das Nachsprechen sowie die Schriftsprache stellten sich zum Untersuchungszeitpunkt unauffällig dar (Tab. 1). Eine Sprechapraxie oder Dysarthrie bestand nicht. Bei Durchführung des AAT hatte schon eine beginnende Rekonstitution eingesetzt, die Punktwerte (Prozentränge) ergaben: Token-Test 0 (99), Nachsprechen 148 (97), Schriftsprache 90 (100), Benennen 110 (94), Sprachverständnis 106 (93).

**Table 1:** 

Fall	Sprachverständnis	Flüssigkeit	Syntax	Schriftsprache	Nachsprechen	Benennen auf Satzebene
1	Gestört auf Satzebene	Nichtflüssig	Vereinfacht	Initial leichtgradig eingeschränkt	Unauffällig	Gestört
2	Gestört auf Satz- und Wortebene	Nichtflüssig	Vereinfacht	Leichtgradig eingeschränkt	Satzteilauslassungen	Gestört
3	Gestört auf Satzebene	Nichtflüssig	Vereinfacht	Initial leichtgradig eingeschränkt	Unauffällig	Gestört

### Fall 2

Eine 80-jährige Patientin stellte sich aufgrund einer seit dem Vortag bemerkten Verlangsamung der Sprachproduktion vor. In der erweiterten Anamnese wurden vorbestehende sprachliche oder anderweitige kognitive Defizite seitens des Ehemanns verneint. MR-tomographisch fand sich eine Diffusionsrestriktion in der linken Capsula externa, die in das frontale periventrikuläre Marklager reichte und dort das Corpus nuclei caudati erreichte (Abb. 4, Patientin 2).

In der logopädischen Untersuchung zeigte sich ein reduziertes auditives Sprachverständnis auf Wort- und Satzebene mit im Vordergrund stehenden semantischen Fehlern. Die Spontansprache war durch einen stockenden Redefluss mit Wortfindungsstörungen und Interjektionen sowie durch Suchverhalten, mit aber letztlich erfolgreichem Wortabruf, geprägt. Die Spontansprache war syntaktisch vereinfacht, jedoch ohne grammatikalische Fehler. Das Benennen auf Wortebene zeigte syntagmatische Paraphasien (Wortumschreibungen wie z. B. „Behälter für Briefe“ anstatt „Briefkasten“) und den Gebrauch von Floskeln; auch auf Satzebene war das Benennen von Aktionen gestört. Das Nachsprechen war erst auf Satzebene beeinträchtigt, wobei es vorwiegend zu Auslassungen von Satzteilen kam. Die Schriftsprache war leichtgradig eingeschränkt mit vereinzelten Paragraphien (Buchstabenverwechslungen; Tab. 1). Eine Sprechapraxie oder Dysarthrie lag nicht vor. Der AAT mit Punktwerten (Prozenträngen) fiel wie folgt aus: Token-Test 9 (83), Nachsprechen 139 (84), Schriftsprache 81 (90), Benennen 95 (72), Sprachverständnis 97 (79). Nebenbefundlich ergaben sich in einer neuropsychologischen Kurztestung Hinweise auf kognitive Defizite (Mini Mental State 23/30, Demtect 4/18 Punkten), welche fremdanamnestisch nicht vorbekannt gewesen seien.

### Fall 3

Eine 64-jährige Patientin stellte sich vor, da sie sowohl undeutlich als auch stockend sprechen würde. Zusätzlich sei ihr ein Schweregefühl des rechten Armes aufgefallen. Die Symptome hätten zwei Tage vor der Vorstellung begonnen. In der durchgeführten cMRT konnte ein linksseitiger lakunärer Infarkt mit Beteiligung des kaudalen Putamens und Ausbreitung in die Capsula externa nachgewiesen werden (Abb. 4, Patientin 3).

In der logopädischen Untersuchung zeigte sich das Sprachverständnis auf Satzebene geringgradig eingeschränkt. Darüber hinaus ließ sich neben einer paretischen Dysarthrie eine nichtflüssige Spontansprache mit vereinfachter Syntax ohne grammatikalische Fehler mit leichten Wortfindungsstörungen und Interjektionen, schlussendlich aber korrektem Wortabruf, abgrenzen. Beim Benennen waren die Sätze vereinfacht und inhaltlich ungenau, das Benennen auf Wortebene war hingegen unauffällig. Das Nachsprechen zeigte keine Einschränkungen. Die Schriftsprache war nur unmittelbar bei Aufnahme mit einzelnen Buchstabenverwechslungen auffällig (Tab. 1). Im AAT ließen sich folgende Punktwerte (Prozentränge) nachweisen: Token-Test 0 (99), Nachsprechen 149 (99) Schriftsprache 90 (100), Benennen 110 (94), Sprachverständnis 112 (98).

### Nichtaphasischer Kontrollpatient (Fall 4)

Ein 51-jähriger Patient bemerkte am Morgen des Vorstellungstages nach dem Erwachen eine leichtgradige Hemiparese rechts sowie eine milde Dysarthrie. Beim Eintreffen zeigte sich die Symptomatik bereits zunehmend rückläufig. Logopädisch wurde eine Aphasie oder Sprechapraxie ausgeschlossen. In der cMRT zeigte sich ein linksseitiger, subakuter Infarkt im kraniodorsal der Capsula interna gelegenen Marklager mit Angrenzung an das dorsale Putamen (Abb. 4, Patient 4).

#### Zusammenfassend

zeigte sich in der logopädischen Untersuchung in den ersten drei Fällen eine leicht- bis mäßiggradige nichtflüssige Aphasie mit vereinfachter Syntax in der Spontansprache und beim Benennen sowie eine Verständnisstörung für komplexe Syntax (Tab. 1). Inkonstant fanden sich Probleme beim Nachsprechen und eine Dysarthrie.

Bildmorphologisch zeigte sich in allen drei Fällen eine weit lateral gelegene Lakune mit Beteiligung der linken Capsula externa und Ausbreitung bis in das periventrikuläre frontale Marklager. Die Lakune des nichtaphasischen Kontrollpatienten lag im Vergleich zu den Lakunen der aphasischen Patienten weiter ventrokaudal (Abb. 1 und 4), wobei die Lakune der Patientin 3 die größte Schnittmenge mit der Lakune des nichtaphasischen Kontrollpatienten aufwies.

Die Fasertraktographie in den Gehirnen zweier gesunder Probanden, ausgehend von den Orten der Lakunen der drei aphasischen Patienten und der Lakune des Kontrollpatienten, zeigte bei allen Patienten eine Betroffenheit des linken Fasciculus longitudinalis superior sowie bei den aphasischen Patienten eine deutliche Beteiligung des linken Fasciculus arcuatus (Abb. 2). Zusätzlich fand sich bei den aphasischen Patienten eine Affektion des frontostriatalen Trakts und des frontalen Aslant-Trakts. Transkallosale sowie weiter absteigende Fasern in die Capsula interna waren vereinzelt nachweisbar, ohne ein konsistentes Muster zu zeigen (Abb. 2). Mittels probabilistischer Fasertraktographie fanden sich bei den aphasischen Patienten kortikale Projektionen im Bereich des temporalen Kortex sowie des Gyrus frontalis inferior mit hauptsächlicher Projektion in die Pars triangularis und opercularis (Abb. 3a, b). Bei dem nichtaphasischen Patienten projizierten die verfolgbaren Fasern entsprechend der Zielregionen des Fasciculus longitudinalis superior in den präfrontalen und parietalen Kortex sowie in den Gyrus praecentralis (Abb. 3a, b). Fasern aus dem Teil der Lakune von Patientin 3, der der Lakune des nichtaphasischen Patienten am nächsten gelegen, jedoch nicht überlappend war, projizierten in die Pars triangularis und opercularis des inferioren frontalen Gyrus und in die anteriore Insel (Abb. 3c, d).

## Diskussion

Wir identifizierten bei drei Patienten mit lakunären Hirninfarkten eine leicht- bis mäßiggradige expressiv betonte Aphasie mit im Vordergrund stehender Reduktion der Sprachflüssigkeit und der syntaktischen Komplexität.

Die Lakunen der aphasischen Patienten lagen lateral in der Capsula externa und betrafen sowohl Fasern des Fasciculus arcuatus und Fasciculus longitudinalis superior als auch des frontostriatalen und frontalen Aslant-Trakts. Die letztgenannten Trakte, die präsupplementär motorische Areale im Interhemisphärenspalt mit dem Nucleus caudatus sowie dem linken inferioren frontalen Gyrus verbinden, sind vor allem mit Sprachinitiierung in Verbindung gebracht worden [21]. Die subkortikale elektrische Stimulation dieser Fasertrakte zeigte eine der bei unseren Patienten beobachteten Aphasie nicht unähnliche Symptomatik. Ebenfalls sind aphasische Symptome bei Läsionen des Fasciculus arcuatus und des Fasciculus longitudinalis superior beschrieben worden, wobei diese vor allem Defizite beim Nachsprechen beinhalteten, die bei unseren Patienten nicht im Vordergrund standen. Defizite beim Nachsprechen werden v. a. mit kaudalen Läsionen dieser Trakte in Verbindung gebracht [22]. Rostrale, aber nicht kaudale Läsionen dieser longitudinalen Trakte gehen ebenso mit einer reduzierten Sprachflüssigkeit in der Spontansprache einher [10], was durch eine gleichzeitige Schädigung der absteigenden Faserbahnen in diesem Bereich erklärt werden könnte. Während die Lakunen der aphasischen Patienten konsistent absteigende Faserbahnen aus prämotorischen Arealen betrafen, zeigte die Kontrolllakune eines nichtaphasischen Patienten in unmittelbarer Nachbarschaft eine ventrokaudalere Lokalisation mit Betroffenheit kaudalerer absteigender Fasern inklusive der Pyramidenbahn. Die Bedeutung der Lokalisation der Läsion und damit der Betroffenheit von Faserbahnen erinnert an das Konzept strategischer Lakunen bei der Entwicklung subkortikaler Demenzen im Rahmen einer zerebralen Mikroangiopathie. Hierbei spielt insbesondere die strategische Lokalisation der lakunären Infarkte mit Affektion von Fasertrakten, die in den präfrontalen Kortex ziehen, oder mit Betroffenheit grauer Substanz wie des Thalamus eine entscheidende Rolle [7]. Die Auffälligkeiten in der kognitiven Testung von Patientin 2 werteten wir jedoch am ehesten bedingt durch die Abhängigkeit solcher Tests von intakten Sprachleistungen.

Wir können nicht ausschließen, dass jenseits der Läsionen weißer Substanz auch Läsionen subkortikaler grauer Substanz zur Aphasie beigetragen haben, da vereinzelt das Pallidum oder der Nucleus caudatus mit in die Lakune einbezogen waren. Konsistente Läsionen der Basalganglien fanden sich jedoch in den beschriebenen Fällen nicht.

Auch wenn subkortikale Sprachstörungen im Vergleich zu kortikalen Aphasien meist milder ausgeprägt sind, handelt sich hierbei dennoch um alltagsrelevante Einschränkungen [20]. Trotz einer vorherrschenden Unsicherheit bezüglich der Wirksamkeit einer intravenösen Thrombolyse bei lakunären Infarkten sollte daher, falls möglich, nicht auf diese verzichtet werden. Diesbezüglich ist erwähnenswert, dass eine rezente Subgruppenanalyse der WAKE-UP-Studie eine Verbesserung des funktionellen Outcomes nach intravenöser Thrombolyse auch bei lakunären Hirninfarkten nachgewiesen hat [3].

Zusammenfassend können Läsionen der in der Capsula externa laufenden Faserbahnen durch eine sehr weit lateral gelegene Lakune zur Ausbildung einer mäßiggradigen Aphasie führen. Der Schädigungsort betrifft eine Region weißer Substanz, in der sowohl der frontostriatale Trakt, der frontale Aslant-Trakt, aber auch der Fasciculus arcuatus verlaufen. Die Koinzidenz dieser drei Fälle innerhalb kurzer Zeit schärfte unseren Blick für dieses atypische lakunäre Syndrom.

## Fazit für die Praxis

- Aphasien können in seltenen Fällen auch auf lakunäre Hirninfarkte zurückzuführen sein.
- Läsionen im Bereich der linken Capsula externa, in der der Fasciculus arcuatus sowie der frontostriatale und frontale Aslant-Trakt verlaufen, können einer lakunären Aphasie zugrunde liegen.
- Aufgrund der alltagsrelevanten funktionellen Beeinträchtigung sollte eine Thrombolysetherapie im 4,5-h-Zeitfenster erwogen werden.
